# Supplementary material for: HIVprotI: an integrated web based platform for prediction and design of HIV proteins inhibitors
Source: J Cheminform. 2018 Mar 9;10:12. doi: 10.1186/s13321-018-0266-y (PMC5845081; doi:10.1186/s13321-018-0266-y)
Supplement: Supplementary file 2 — Additional file 2. Source code of HIVProtI web server. [file 13321_2018_266_MOESM2_ESM.zip › HIVprotI_Source-code/hivprotI/weka-3-6-6/doc/weka/classifiers/CheckSource.html]

CheckSource


---


|  |  |  |
| --- | --- | --- |
| **Overview** \| **Package** \| **Class** \| **Tree** \| **Deprecated** \| **Index** \| **Help** \| **Weka's home** | |  |
| **PREV CLASS**   **NEXT CLASS** | **FRAMES**    **NO FRAMES**     **All Classes** |  |
| SUMMARY: NESTED \| FIELD \| CONSTR \| METHOD | DETAIL: FIELD \| CONSTR \| METHOD |  |


---


## weka.classifiers Class CheckSource

```
java.lang.Object
  weka.classifiers.CheckSource
```

**All Implemented Interfaces:**: OptionHandler, RevisionHandler

---

``` public class CheckSource extends java.lang.Object implements OptionHandler, RevisionHandler ```

A simple class for checking the source generated from Classifiers
implementing the `weka.classifiers.Sourcable` interface.
It takes a classifier, the classname of the generated source
and the dataset the source was generated with as parameters and tests
the output of the built classifier against the output of the generated
source. Use option '-h' to display all available commandline options.
Valid options are:

```
 -W <classname and options>
  The classifier (incl. options) that was used to generate
  the source code.
```

```
 -S <classname>
  The classname of the generated source code.
```

```
 -t <file>
  The training set with which the source code was generated.
```

```
 -c <index>
  The class index of the training set. 'first' and 'last' are
  valid indices.
  (default: last)
```

Options after -- are passed to the designated classifier (specified with -W).

**Version:**
:   $Revision: 1.4 $

**Author:**
:   fracpete (fracpete at waikato dot ac dot nz)

**See Also:**: `Sourcable`

---

**Constructor Summary**

`CheckSource()`


| **Method Summary** | |
| --- | --- |
| `boolean` | `execute()`             performs the comparison test |
| `Classifier` | `getClassifier()`             Gets the classifier being used for the tests, can be null. |
| `int` | `getClassIndex()`             Gets the class index of the dataset. |
| `java.io.File` | `getDataset()`             Gets the dataset to use for testing, can be null. |
| `java.lang.String[]` | `getOptions()`             Gets the current settings of the Classifier. |
| `java.lang.String` | `getRevision()`             Returns the revision string. |
| `Classifier` | `getSourceCode()`             Gets the class to test. |
| `java.util.Enumeration` | `listOptions()`             Returns an enumeration describing the available options. |
| `static void` | `main(java.lang.String[] args)`             Executes the tests, use "-h" to list the commandline options. |
| `void` | `setClassifier(Classifier value)`             Sets the classifier to use for the comparison. |
| `void` | `setClassIndex(int value)`             Sets the class index of the dataset. |
| `void` | `setDataset(java.io.File value)`             Sets the dataset to use for testing. |
| `void` | `setOptions(java.lang.String[] options)`             Parses a given list of options. |
| `void` | `setSourceCode(Classifier value)`             Sets the class to test. |

**Methods inherited from class java.lang.Object**

`equals, getClass, hashCode, notify, notifyAll, toString, wait, wait, wait`

**Constructor Detail**

### CheckSource

```
public CheckSource()
```


**Method Detail**

### listOptions

```
public java.util.Enumeration listOptions()
```

:   Returns an enumeration describing the available options.

    :   **Specified by:**: `listOptions` in interface `OptionHandler`
    :   **Returns:**: an enumeration of all the available options.

---


### setOptions

```
public void setOptions(java.lang.String[] options)
                throws java.lang.Exception
```

:   Parses a given list of options.


    Valid options are:

    ```
     -W <classname and options>
      The classifier (incl. options) that was used to generate
      the source code.
    ```

    ```
     -S <classname>
      The classname of the generated source code.
    ```

    ```
     -t <file>
      The training set with which the source code was generated.
    ```

    ```
     -c <index>
      The class index of the training set. 'first' and 'last' are
      valid indices.
      (default: last)
    ```

    Options after -- are passed to the designated classifier (specified with
    -W).

    :   **Specified by:**: `setOptions` in interface `OptionHandler`
    :   **Parameters:**: `options` - the list of options as an array of strings **Throws:**: `java.lang.Exception` - if an option is not supported

---


### getOptions

```
public java.lang.String[] getOptions()
```

:   Gets the current settings of the Classifier.

    :   **Specified by:**: `getOptions` in interface `OptionHandler`
    :   **Returns:**: an array of strings suitable for passing to setOptions

---


### setClassifier

```
public void setClassifier(Classifier value)
```

:   Sets the classifier to use for the comparison.

    :   **Parameters:**: `value` - the classifier to use

---


### getClassifier

```
public Classifier getClassifier()
```

:   Gets the classifier being used for the tests, can be null.

    :   **Returns:**: the currently set classifier

---


### setSourceCode

```
public void setSourceCode(Classifier value)
```

:   Sets the class to test.

    :   **Parameters:**: `value` - the class to test

---


### getSourceCode

```
public Classifier getSourceCode()
```

:   Gets the class to test.

    :   **Returns:**: the currently set class, can be null.

---


### setDataset

```
public void setDataset(java.io.File value)
```

:   Sets the dataset to use for testing.

    :   **Parameters:**: `value` - the dataset to use.

---


### getDataset

```
public java.io.File getDataset()
```

:   Gets the dataset to use for testing, can be null.

    :   **Returns:**: the dataset to use.

---


### setClassIndex

```
public void setClassIndex(int value)
```

:   Sets the class index of the dataset.

    :   **Parameters:**: `value` - the class index of the dataset.

---


### getClassIndex

```
public int getClassIndex()
```

:   Gets the class index of the dataset.

    :   **Returns:**: the current class index.

---


### execute

```
public boolean execute()
                throws java.lang.Exception
```

:   performs the comparison test

    :   **Returns:**: true if tests were successful **Throws:**: `java.lang.Exception` - if tests fail

---


### getRevision

```
public java.lang.String getRevision()
```

:   Returns the revision string.

    :   **Specified by:**: `getRevision` in interface `RevisionHandler`
    :   **Returns:**: the revision

---


### main

```
public static void main(java.lang.String[] args)
                 throws java.lang.Exception
```

:   Executes the tests, use "-h" to list the commandline options.

    :   **Parameters:**: `args` - the commandline parameters **Throws:**: `java.lang.Exception` - if something goes wrong


---


|  |  |  |
| --- | --- | --- |
| **Overview** \| **Package** \| **Class** \| **Tree** \| **Deprecated** \| **Index** \| **Help** \| **Weka's home** | |  |
| **PREV CLASS**   **NEXT CLASS** | **FRAMES**    **NO FRAMES**     **All Classes** |  |
| SUMMARY: NESTED \| FIELD \| CONSTR \| METHOD | DETAIL: FIELD \| CONSTR \| METHOD |  |


---
